# Our New York Letter

**Published:** 1889-01

**Authors:** 


### Correspondence.

### OUR NEW YORK LETTER.

THE AMERICAN ACADEMY OF MEDICINE—THE OPIUM HABIT—POST-GRADUATE INSTRUCTION—TOLERATION AND INTOLERATION IN MEDICINE AND THE CODE OF ETHICS—THE MEDICO-LEGAL SOCIETY ON ELECTRICAL EXECUTION OF CRIMINALS—DEATH OF DR. H. B. SANDS—THE GERMAN HOSPITAL—THE TRAINING SCHOOLS FOR MALE NURSES.

*To the Editors Atlanta Medical and Surgical Journal:*

The twelfth annual session of the American Academy of Medicine was held at the New York Hospital, in that part of the institution known as the Governor's room. The morning meeting began with about sixty members present, Dr. F. H. Gerrish, of Portland, Me., the President, occupying the chair. After an election of fifty new members, Dr. J. C. Wilson, of Philadelphia, read a paper on the opium habit. He said, among other things, that patients taking opium should be kept in ignorance of the fact, and that druggists should not renew prescriptions for this drug without an order from the doctor. About forty attended the afternoon session. The President's address, in which he spoke particularly of the necessity of thoroughly educating medical students, was delivered at this session. Medical Colleges, he said, should require a preliminary examination. A number of interesting papers were then read, and in the evening the members went to Martinelli's, where a dinner was served. The reading of a long list of toasts, and responding to them, ended the programme of the Academy for the first day. On the second day the attendance fell off somewhat. Among the most important topics discussed was the subject of post-graduate instruction. Dr. C. C. Lee, of New York, in an address, remarked that New York had the first school of this kind, while at present there are six in the United States. He said that no city in the Old World possessed greater clinical advantages than New York did just now, but, at the same time, he recommended medical men to visit Europe, as it tended to make one more liberal in his ideas, and new methods would be learned. A very heated discussion followed Dr. Bowditch's paper on "Toleration and Intoleration in Medicine and Code of Ethics." His resolution, to the effect that the chasm existing between the two divisions was detrimental to both sides, and that closer relations would enhance their mutual interests, was hotly debated, and finally laid on the table. After some other papers were read, the Academy adjourned to meet next year in Chicago.

At the last monthly meeting of the Medico-Legal Society, held at the Buckingham Hotel, a report of the committee appointed to consider and advise upon the proper method of executing criminals by electricity was presented. It is a long article, and goes considerably into details, concluding as follows: "After mature deliberation, we recommend that the death-current be administered to the criminal in the following manner: A stout table covered with rubber cloth, and having holes along its borders for binding, or a strong chain should be procured; the prisoner, lying on his back or sitting, should be firmly bound upon this table or in the chair. One electrode should be so inserted into the table or into the back of the chair, that it will impinge upon the spine between the shoulders. The head should be secured by means of a sort of helmet, fastened to the table or back of the chair, and to this helmet the other pole should be so joined as to press firmly with its end upon the top of the head. We think a chair is preferable to a table. The rheophores can be led off to the dynamo through the floor or to another room, and the instrument for closing the circuit can be attached to the wall. The electrodes should be of metal, not over one inch in diameter, somewhat ovoid in shape, and covered with a thick layer of sponge or chamois skin. The poles and the skin and hair at the points of contact, should be thoroughly wet with warm water. The hair should be cut short. A dynamo generating an electro-motive force of at least 3,000 volts should be employed. Either a continuous or an alternating current may be used, but preferably the latter. The current should be allowed to pass for thirty seconds." The discussion on the above report will not occur until the next meeting of the society.

The very sudden death of Dr. Henry B. Sands was a profound shock to nearly all who knew him, although there are some physicians who are never surprised by the sudden death of a person over forty. While riding in his carriage, in his usual state of good health, and accompanied by his friend, Dr. A. A. Smith, he suddenly died. Although an autopsy was made by several eminent pathologists, no visible lesions were found to account for this occurrence, and the unsatisfactory cause known as "cardiac failure" had to be given. Dr. Sands was in his fifty-ninth year, and fully deserved his reputation of being the first surgeon in New York. He had lately retired from hospital duties, but retained his professorship in the college, and also enjoyed the largest surgical consulting practice in the city. His funeral was largely attended, and among those present were about three hundred students from the College of Physicians and Surgeons. The eulogy was delivered by Dr. W. M. Taylor, who truthfully remarked that Dr. Sands was "the idol of his profession," and, also, that "he was great in all he did."

The German Hospital celebrated the opening of a new building a few nights ago, in a very pleasant way. Addresses were made by Dr. F. Lange, and others, and music was furnished by the Liederkrantz and Arion Clubs. The new structure is situated on Fourth Avenue, and is three stories high in front, and four in the rear. It is built fire-proof and contains a kitchen, ice-house, doctors' rooms, a large operating-room, and numerous other apartments.

The Bellevue Hospital Training-School for Male Nurses, will in a few days issue a prospectus for all persons intending to enter the department. The requirements will be substantially the same as I have described before. It also states that, while on duty, nurses will have the following uniform: Blue and white seersucker, simply made; plain white apron, cap and linen collar. It is expected that this school will enable us to procure competent male nurses, with much less difficulty than exists at present.

*New York, December* 8, 1888. A. F.

